# A novel radiopaque tissue marker for soft tissue localization and *in vivo* length and area measurements

**DOI:** 10.1371/journal.pone.0224244

**Published:** 2019-10-18

**Authors:** Sambit Sahoo, Andrew R. Baker, Bong Jae Jun, Ahmet Erdemir, Eric T. Ricchetti, Joseph P. Iannotti, Kathleen A. Derwin

**Affiliations:** 1 Department of Biomedical Engineering, Lerner Research Institute, Cleveland Clinic, Cleveland, Ohio, United States of America; 2 Department of Orthopaedic Surgery, Orthopedic and Rheumatologic Institute, Cleveland Clinic, Cleveland, Ohio, United States of America; Stanford University School of Medicine, UNITED STATES

## Abstract

**Purpose:**

The purpose of the study was to describe the characteristics and demonstrate proof-of-concept and clinical use of a barium sulfate infused polypropylene radiopaque tissue marker for soft tissue localization and *in vivo* measurement of lengths and areas.

**Methods:**

Marker mechanical properties were evaluated by tensile tests. Biocompatibility was evaluated following 8–12 weeks’ implantation in a pig model. Proof-of-concept of marker application was performed in a human cadaveric shoulder model, and methods for CT imaging and measurement of dimensions were established. Lastly, the method of clinical use of the markers was described in one patient undergoing arthroscopic rotator cuff repair (RCR).

**Results:**

The radiopaque markers had a tensile strength of 28 ±4.7 N and were associated with minimal to mild inflammatory tissue reaction similar to polypropylene control. CT-based measurements showed relatively high precisions for lengths (0.66 mm), areas (6.97 mm^2^), and humeral orientation angles (2.1°) in the cadaveric model, and demonstrated 19 ±3 mm medio-lateral tendon retraction and 227 ±3 mm^2^ increase in tendon area in the patient during 26 weeks following RCR. No radiographic leaching, calcification or local adverse events were observed.

**Conclusions:**

The radiopaque tissue marker was biocompatible and had adequate strength for handling and affixation to soft tissues using standard suturing techniques. The marker could be used with low-dose, sequential CT imaging to quantitatively measure rotator cuff tendon retractions with clinically acceptable accuracy. We envision the radiopaque tissue marker to be useful for soft tissue localization and *in vivo* measurement of tissue and organ dimensions following surgery.

## Introduction

Accurate and repeatable techniques for radiographic localization and *in vivo* measurement of the dimensions of tissues and organs are desirable in various surgical disciplines. Implantable radiopaque markers have been used in orthopedic applications to monitor the healing/failure of tendon and ligament repairs [1–6]. In particular, radiopaque marker tracking using three-dimensional (3D) X-ray imaging techniques such as radiostereometric analysis (RSA) and computed tomography (CT) has been used to monitor anterior cruciate ligament (ACL) graft stretching and migration [1], stiffness of healing Achilles tendon repairs [2], and length across rotator cuff repairs (RCR) to indicate healing/failure after surgery [3–6]. These techniques permit soft tissue measurements with accuracy <1 mm [1, 7, 8]. Implanted radiopaque markers have also been used to reconstruct the 3D geometry of the heart and track movements of the heart wall.[9–11]. Implanted radiopaque markers have also been used during radiotherapy planning and image-guided treatment to locate tumor margins and internal organ movement in tumors of the prostate [12–15], uterine cervix [16], lung [17], liver, and spinal/paraspinal lesions [18].

A review of the literature reveals that all prior studies [9–18] used metal-based radiopaque tissue markers made from tantalum, platinum, gold, or stainless steel in the form of spherical beads (~0.8–1.6 mm diameter), rods, rings, or sutures. The use of metal markers has been known to be associated with the risk of breakage [5] and migration within soft tissues [3, 8]. Such markers often therefore require securing by a secondary means such as suture fixation [19–22]. While feasible, affixing metal markers to tissues with suture is technically challenging and time-consuming and therefore not broadly applicable outside of the research setting. Hence, an implantable non-metallic radiopaque marker that can be readily affixed to tissues with minimal time or technical burden and allows accurate localization and length measurements would be desirable for radiographic localization and *in vivo* measurement of tissue and organ dimensions in potentially a broad range of surgical applications.

To address this need, we have developed a radiopaque tissue marker that can be readily tied to body tissues during surgical procedures to allow radiographic visualization of the target locations. We have previously demonstrated the application of this marker in a porcine hernia model, wherein the marker was used to objectively assess and quantify hernia formation, localize mesh implants and monitor hernia size and volume using longitudinal CT imaging [23, 24]. In this manuscript, we describe the mechanical properties, biocompatibility and sub-chronic toxicity, proof-of-concept and finally clinical use of the radiopaque tissue marker for soft tissue localization and measurement of tendon retraction following rotator cuff repair. We hypothesize that the radiopaque tissue marker can be readily affixed to rotator cuff tendon using arthroscopic technique and used with CT imaging to quantitatively measure tendon retraction following rotator cuff repair.

## Materials and methods

### Radiopaque tissue marker

The radiopaque tissue marker (FibermarX^™^, Viscus Biologics LLC, Cleveland, OH) is a sterile, USP size-0 polypropylene monofilament infused with barium sulfate. The marker is visible on standard radiographs (e.g., X-ray, mammography, CT) and has been cleared by the US Food and Drug Administration (FDA) for use during open, percutaneous, or arthroscopic/ laparoscopic/ endoscopic procedures to radiographically mark a soft tissue location during a surgical procedure or for future surgical procedures (510(k), K170026).[25]

### Mechanical properties

Tensile tests were conducted using a material testing machine (MTS 858, MTS systems, Eden Prairie, MN) equipped with a 100 lb (~45 kg) load cell affixed to a Capstan fixture following the ASTM D 2256–02 specifications.[26] Segments of the radiopaque marker (30 cm long, n = 3) were affixed to the Capstan fixture and pulled at a constant rate of 30 cm/min until failure. For comparison, segments of USP size-0, 2–0, and 3–0 polypropylene (Prolene^™^, Ethicon) were also tested as a non-barium sulfate loaded, polypropylene controls (n = 3/ group). The breaking strength (maximum load attained by specimen prior to failure) and percent elongation (maximum elongation of the suture divided by the initial gage length, 22.6cm) were determined.

### Biocompatibility and sub-chronic toxicity

Biocompatibility and sub-chronic toxicity of the radiopaque marker were evaluated following 8 and 12 weeks of implantation in the abdominal wall of two female Yorkshire pigs (30-40kg, 3–4 months old, Michael Fanning Farms, Howe, IN) that were part of a separate study investigating hernia repair [23, 24]. At the time of hernia surgery, the radiopaque marker and USP size 2–0 Prolene^™^ markers (as a non-barium sulfate loaded, polypropylene control) were applied on the rectus abdominal musculofascial layer remote to the hernia repair site as interrupted simple sutures secured with a knot with four alternating half-hitches. All animal experiments complied with the National Institutes of Health guide for the care and use of Laboratory animals [27] and were approved by the Institutional Animal Care and Use Committee at the Cleveland Clinic (Approval # ARC 2014–1307). The NC3Rs ARRIVE Guidelines checklist is presented in the S1 Checklist.

Postoperative analgesia was provided by the Fentanyl transdermal patch (50 μg/h, applied pre-operatively), IM buprenorphine (0.005–0.01 mg/kg, 2 doses on day of surgery) and IM meloxicam (5mg OD on day of surgery and the day after, if required). All animals were monitored for postoperative complications during the 12 week course of the study.

Following 8 and 12 week of implantation, the animals (one at each time-point) were anaesthetized using IM ketamine (20 mg/kg) and xylazine (2 mg/kg), and euthanized (0.2ml/kg Beuthanasia, I.V.; Intervet/Merck Animal Health, Madison, NJ). The abdomen was opened and tissue strips containing the radiopaque marker and 2–0 Prolene were harvested, fixed in 10% neutral buffered formalin and processed for routine paraffin embedding and histology. After hematoxylin and eosin (H&E) staining, the histology sections were scanned in their entirety at 20X using a Leica SCN400F scanner (Leica Microsystems, GmbH, Wetzlar, Germany). Representative sections from each animal were descriptively reviewed and assessed for biocompatibility and sub-chronic toxicity by a board-certified pathologist.

### Proof-of-concept assessment in a cadaveric shoulder rotator cuff model

**Fig 1:**
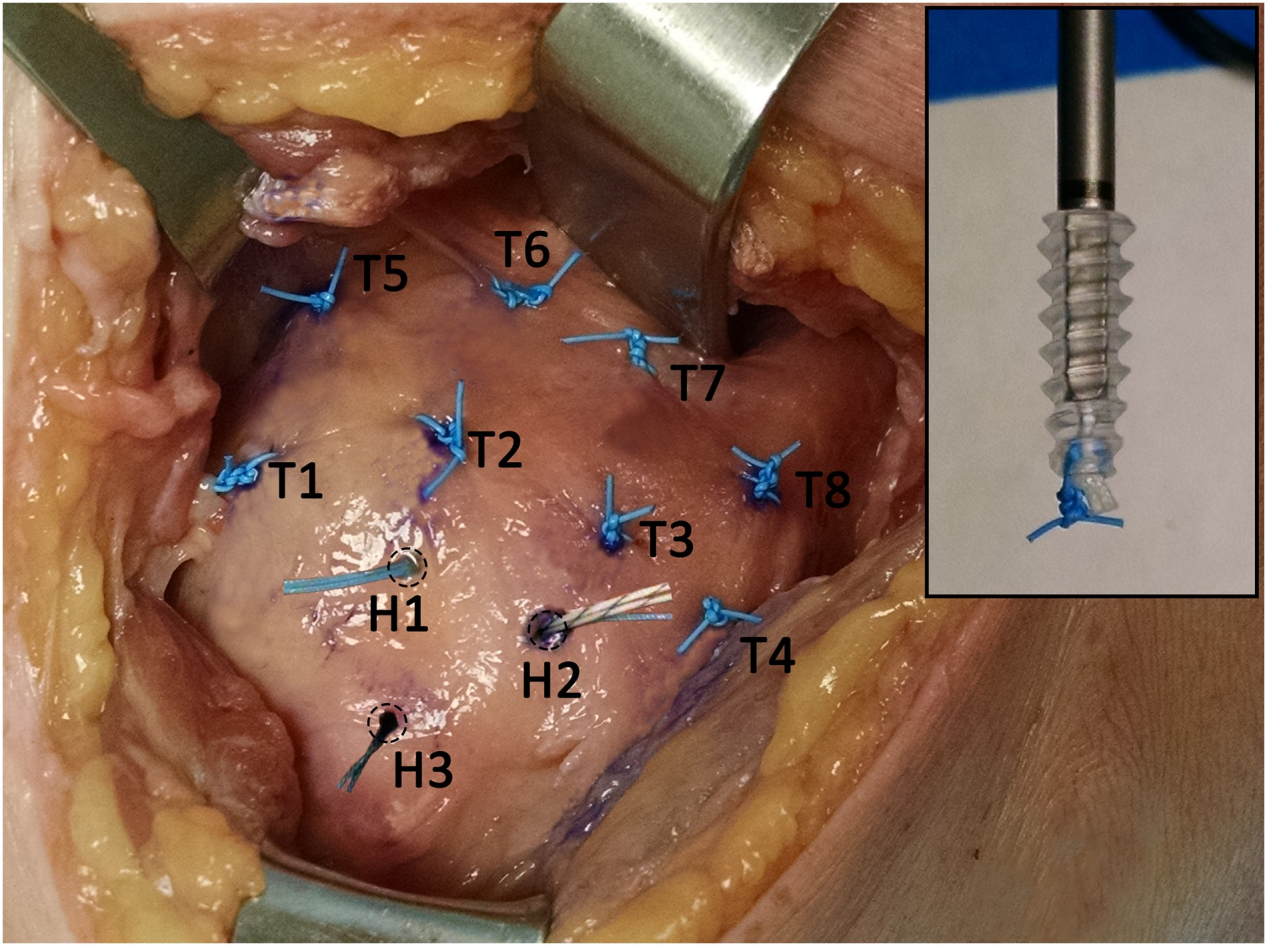
Eight radiopaque tissue markers were secured on the rotator cuff tendons (T1-T8). Three additional markers were tied to the distal tip of suture-anchors (inset) which were secured into the humerus (H1-H3) through the rotator cuff footprint.

To assess the feasibility for clinical use in rotator cuff repair patients, the radiopaque marker was next evaluated in a human cadaveric shoulder model to demonstrate proof-of-concept of marker application, and establish the methods for CT imaging and analysis. A full upper extremity and shoulder girdle from a 75-year-old female donor (Anatomic Gifts Registry, Hanover, MD) was used. The cadaver donor was not from a vulnerable population and the donor or next of kin provided written informed consent that was freely given. The bursal surface of the rotator cuff tendons was exposed incising and retracting the skin and deltoid muscle. After removing any loose bursal tissue to clearly identify the rotator cuff tendons, eight radiopaque markers were applied on the bursal surface of the tendons (T1-T8) as interrupted simple sutures secured with a knot with four alternating half-hitches (Fig 1). The first row of four markers (T1-T4) were placed approximately 1.5 cm from the lateral edge of the tendon footprint, and the second row of four markers (T5-T8) were placed approximately 1 cm medial to the first row. Next, three bone markers (H1-H3) were placed by tying a radiopaque marker knot stack to the distal tip of three bone anchors (Arthrex, BioComposite Corkscrew^®^ FT suture anchors, 5.5 mm) (Fig 1, **inset**). These anchors were subsequently secured into the humerus at the rotator cuff footprint. Next, the deltoid was repositioned and the skin was closed in layers using routine surgical techniques.

The cadaver shoulder was approximated to a neutral position, and imaged on a Siemens SOMATOM Definition Edge scanner having 64 detectors with 128 channels. A low-dose CT protocol (100 kV, 45 mAs, 0.6 mm collimation width; CTDIvol 1.8 mGy) was used to scan the entire specimen. The specimen was then removed from the CT scanner, manipulated and re-approximated to the neutral position, and imaged. This was repeated to obtain a total of three scans for the specimen.

**Fig 2:**
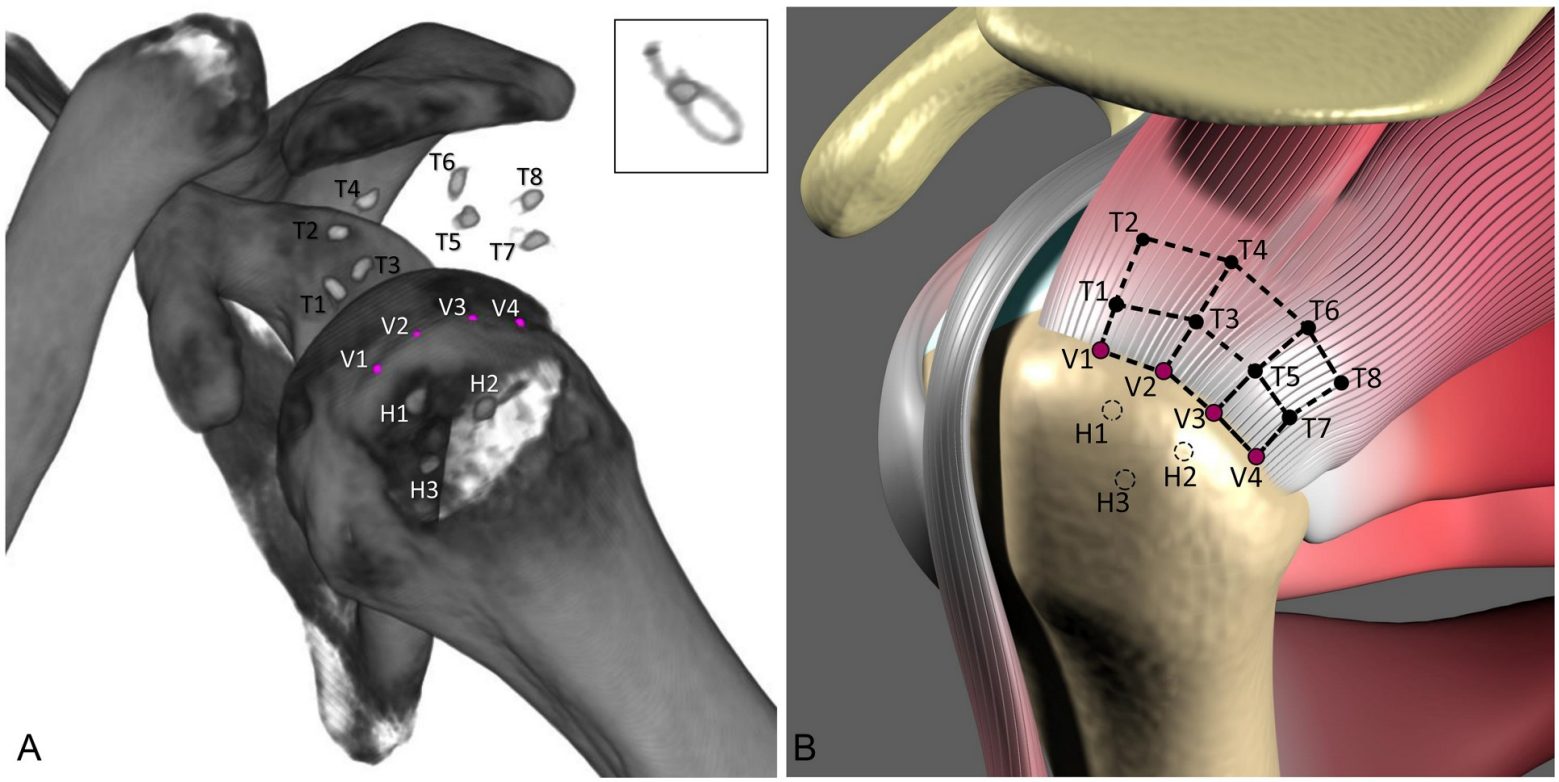
(**A**) CT image of a human cadaver shoulder obtained using a low dose scan protocol (100 kV, 45 mAs, CTDIvol of 1.8 mGy, 0.6mm collimation width), and (**B**) a schematic showing the locations of the radiopaque markers on the rotator cuff tendons (T1-T8) and in the humerus (H1-H3). Four virtual markers were placed on the rotator cuff footprint during image analysis (pink, V1-V4). While the radiopaque marker knot stacks were used to localize the tendon and bone markers, single strands of the marker could also be visualized (inset in A). The 3D coordinates of the tendon and virtual markers were used to calculate select lengths and areas.

Image reconstruction was performed using B40F (soft tissue) convolution kernels without overlap or dose modulation, using a field of view (FOV) of 25.6 cm, and slice-thicknesses of 0.6mm. The CT image sets were analyzed using custom-written image analysis software to determine clinically relevant tendon lengths, areas and humeral orientation. First, the scapular [28–30] and humeral coordinate systems were defined by bony landmarks on the scapula and humerus, respectively, and positive direction was assigned to anterior, superior, and lateral, pointing outward. Next, the eight tendon markers (T1–T8) and three humeral bone markers (H1–H3) were identified by the analyst using the software. The software fit each tissue marker to the volumetric centroid of the voxels identified as a marker. Additionally, four “virtual” bone markers (V1-V4, Fig 2A and 2B) were placed along the lateral edge of the rotator cuff footprint by the analyst, to allow clinically-relevant measurements of displacement of tendon markers with respect to the rotator cuff tendon insertion into the bone.

The 3D coordinates of the tendon, humeral and virtual bone markers computed by the software were analyzed using custom MATLAB code (Mathworks, Natick, MA) to calculate select lengths and the areas enclosed by the FibermarX^™^ marker array (Fig 2B). Briefly, lengths were defined as the Euclidean distance (straight line distance) between markers, and the areas of the quadrilaterals formed by sets of four markers were approximated by determining the sum of the areas of piecewise triangles, i.e., for each triangle using the one half of the magnitude of the cross-product of vectors forming the two edges of the triangle. The area calculation was performed twice and averaged, by swapping the triangulation.

Additionally, humeral orientations relative to the scapular coordinate system were also calculated. Abduction/adduction was defined by the projected angle to the coronal plane between the superior-inferior axes of the humerus and the scapula. Internal/external rotation was defined by the projected angle to the axial plane between the medial-lateral axes of the humerus and the scapula. Flexion/extension was defined by the projected angle to the sagittal plane between the superior-inferior axes of the humerus and the scapula. The mean and standard deviation of lengths, areas and humeral orientation angles were measured for the three repeat scans. The precision of measurements for lengths, areas and humeral orientation angles was estimated by averaging the standard deviations of the respective measures (14 lengths, 6 areas, and 3 angles).

### Radiopaque marker use in patients with rotator cuff repair

The radiopaque marker is currently being used in a prospective clinical study investigating the natural history of healing following arthroscopic rotator cuff repair [31]. 125 patients between 18–75 years, having a 1–5 cm tear of their supraspinatus and/or infraspinatus tendons that is fully repairable by a double row technique, are included in this study conducted under the approval of the Cleveland Clinic Institutional Review Board (Approval # 16–089). Herein, the method of use of the radiopaque marker and preliminary results of CT analysis from one patient (66 year old, female, 2.5 cm tear in supraspinatus tendon) is described to demonstrate proof-of-concept in an *in vivo* setting.

During the tendon repair for this patient, three tantalum beads were introduced into the bone anchor holes just prior to insertion of the anchors, and these serve as the bone markers (H1, H2, H3). Tantalum beads were used as bone markers because the radiopaque marker is currently FDA-cleared only for marking “soft tissue during a surgical procedure or for future surgical procedures” (510(k), K170026).[25] Following tendon repair, four radiopaque markers were tied to the superficial surface of their tendon just medial to the repair sutures at approximately 0.5-1cm intervals in the anterior-posterior direction, using a standard suture lasso and arthroscopic knot tying technique (T1, T3, T5, and T7). The full grid of tendon markers used to demonstrate proof-of-concept in the cadaver study is not used in the clinical study. Multiple postoperative outcomes were measured, but in particular, this patient underwent low-dose CT imaging (100 kV, 45 mAs, 0.6 mm collimation width; 1.8 mGy CTDIvol) at day of surgery, 3 weeks, 12 weeks, and 26 weeks postoperatively to measure tendon retraction using the implanted radiopaque markers. For CT scanning, patients were supine with the affected arm at the side in neutral rotation, and hand on the thigh; the unaffected arm was extended over the head.

The longitudinal CT image sets from the patient were then analyzed by the software described in the previous section to evaluate changes in tendon lengths, areas and humeral orientation over time. The follow-up CT image sets were imported into the software and registered with respect to the scapula from the day of surgery CT image set. For each CT image set, the tendon markers and humeral bone markers were identified and defined by the analyst, and the software computed their 3D coordinates. The coordinates of the four virtual bone markers (V1-V4) are directly imputed from their rigid body relationship to the humeral bone markers established in the reference CT image set. Tendon lengths, areas and humeral orientation are then calculated as described in the previous section, to assess their change over time.

## Results

### Mechanical properties

The radiopaque marker had a tensile strength of 28 ±4.7 N and an elongation at break of 19 ±3.7%. Similar gage USP size-0 Prolene^™^ had a tensile strength of 56 ±0.6 N and an elongation at break of 27 ±0.2%. The mechanical properties of USP size 2–0 and 3–0 Prolene^™^ are also reported for comparison (Table 1).

**Table 1:** Mechanical properties of the radiopaque marker (FibermarX^™^) and non-radiopaque polypropylene (n = 3/group).

	FibermarX^™^	0 Prolene^™^	2–0 Prolene^™^	3–0 Prolene^™^
Tensile Strength (N)	28 ±4.7	56 ±0.6	42 ±1.6	23 ±0.2
Extension at break (%)	19 ±3.7	27 ±0.2	25 ±2.8	31 ±0.5

### Biocompatibility and sub-chronic toxicity

**Fig 3:**
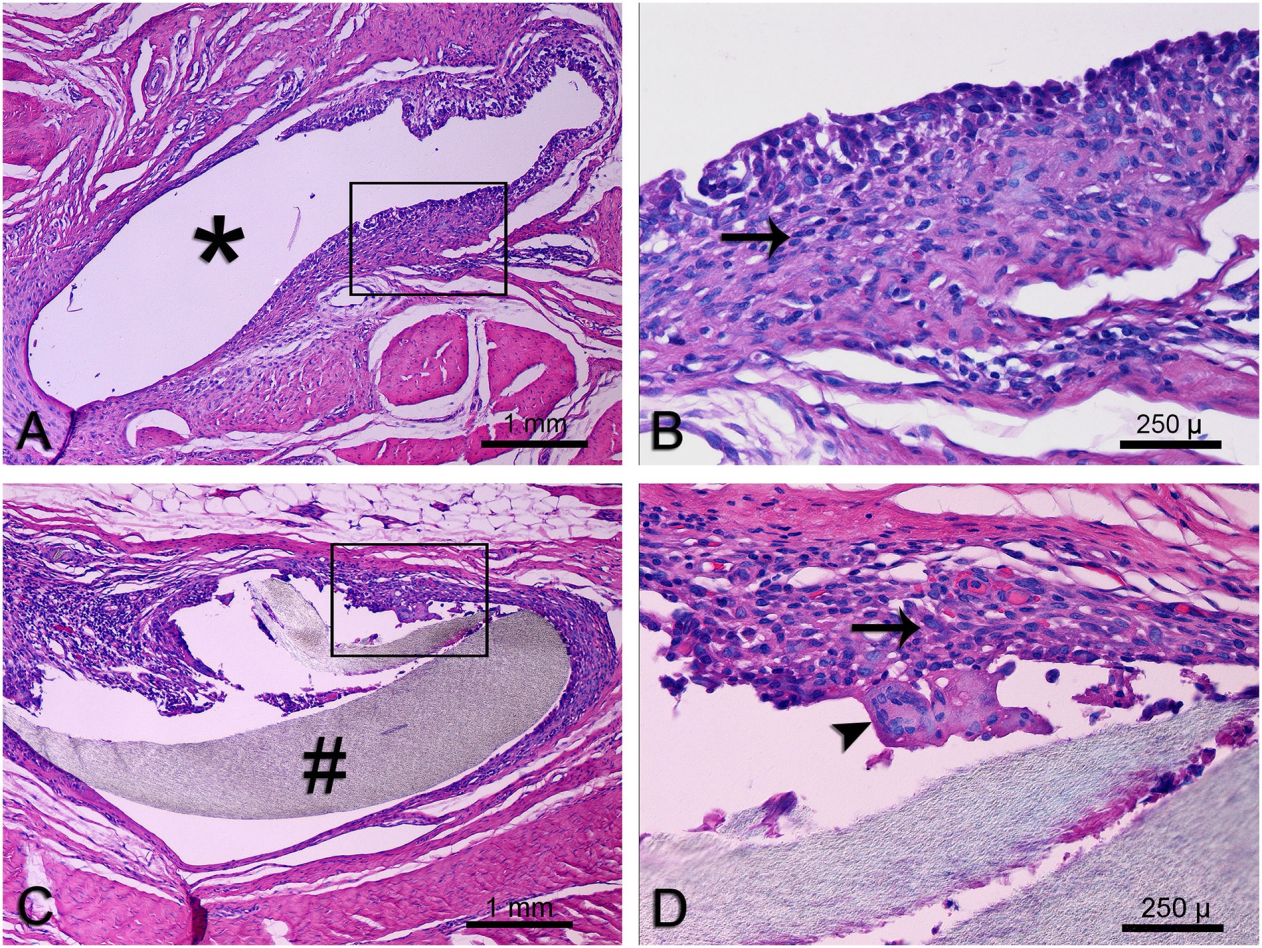
Photomicrographs of representative hematoxylin and eosin-stained sections of the host tissue response to 2–0 Prolene^™^ suture (A, B) and the radiopaque marker (C, D). B and D represent magnified views of the boxed region in A and C respectively; location of 2–0 Prolene^™^ suture (*), radiopaque marker (#), lymphocytes and macrophages (arrows), and a multinucleated giant cell (arrowhead). No difference was observed between the host tissue response to the radiopaque marker and 2–0 Prolene^™^ suture (control).

No difference was observed between the host tissue response to the radiopaque marker and 2–0 Prolene^™^ suture (control) following implantation in the porcine ventral abdominal wall for 12 weeks (Fig 3). The inflammatory cells around the radiopaque marker and Prolene^™^ suture consisted primarily of lymphocytes and macrophages with occasional multinucleated giant cells; eosinophils were absent.

### Feasibility assessment in cadaveric shoulder rotator cuff model

The radiopaque tissue markers could be readily affixed at the desired locations on the rotator cuff tendon in the human cadaver model using standard open suturing techniques. The 4-throw knot portion of the marker was prominent and used for localization by CT image analysis, however, the single pass of the radiopaque marker through the tissue at each tendon location was also readily visualized. As a demonstration of proof-of-concept of using the method for quantitative measurements, select tendon lengths (n = 14) and areas (n = 6) enclosed by the radiopaque tissue marker array, as well as the humeral orientation angles from three CT scans are reported in Table 2. Tendon lengths ranged from 4.4 to 18.3 mm, tendon areas ranged from 61.2 to 215.5 mm^2^, and humeral orientation angles ranged from 14° to 22°. The precision of measurements was 0.66 mm for tendon lengths, 6.97 mm^2^ for tendon areas, and 2.1° for humeral orientation angles.

**Table 2:** Measurements of tendon lengths, areas, and humeral orientation in the cadaveric shoulder rotator cuff model. Data are presented as mean ± standard deviation (SD) of measurements made from three CT scans repeated after manual manipulation and repositioning of the specimen.

Measurement variable		Mean ± SD	Precision
**Tendon, mediolateral lengths (mm)**	**V1T1**	13.2 ± 0.59	0.66
	**T1T2**	10.0 ± 0.93	
	**V2T3**	12.6 ± 0.82	
	**T3T4**	18.2 ± 0.69	
	**V3T5**	14.0 ± 0.45	
	**T5T6**	12.0 ± 0.65	
	**V4T7**	12.4 ± 0.71	
	**T7T8**	10.7 ± 0.42	
**Tendon, antero-posterior lengths (mm)**	**T1T3**	4.4 ± 0.60	
	**T3T5**	18.3 ± 0.55	
	**T5T7**	11.5 ± 0.39	
	**T2T4**	9.6 ± 0.93	
	**T4T6**	13.3 ± 0.86	
	**T6T8**	12.0 ± 0.72	
**Tendon marker areas (mm**^**2**^**)**	**V1V2T3T1**	71.2 ± 1.81	6.97
	**T1T2T3T4**	61.2 ± 5.56	
	**V2V3T5T3**	168.6 ± 5.74	
	**T3T5T6T4**	215.5 ± 19.64	
	**V3V4T7T5**	126.0 ± 4.17	
	**T5T7T8T6**	129.7 ± 4.88	
**Humeral orientation (degrees)**	**Extension**	16 ± 2.1	2.1
	**External rotation**	14 ± 0.9	
	**Abduction**	22 ± 3.3	

### Clinical application in rotator cuff repair patients

**Fig 4:**
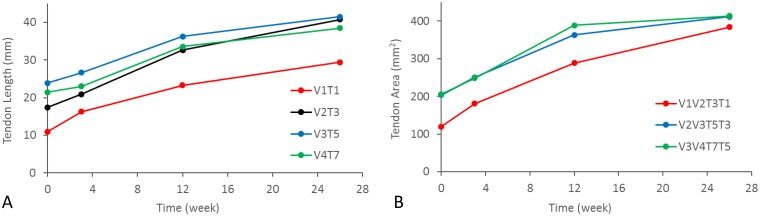
Results from longitudinal analysis of CT data from one patient, for demonstrative purposes, depict an increase in (A) medio-lateral lengths (V1T1, V2T3, V3T5, and V3T7) and (B) areas (V1V2T3T1, V2V3T5T3, and V3V4T7T5) across the rotator cuff tendon repair over 26 weeks post-operation.

The radiopaque tissue markers could be readily affixed intraoperatively at the desired locations on the repaired rotator cuff using standard arthroscopic suturing techniques. No local adverse events were observed in the patient during 26 weeks of follow-up. The knotted portion of the markers was prominent at all time-points, with no evidence of barium leaching or calcification around the markers. Since four lateral row tendon markers were applied in this patient, four medio-lateral tendon lengths, three antero-posterior tendon lengths and three tendon areas enclosed by the radiopaque marker array were calculated, in addition to the humeral orientation angles at the different time-points (Table 3). Fig 4 illustrates the changes in medio-lateral lengths and areas on the rotator cuff tendon during 26 weeks following repair. The interpretation of this finding is part of our ongoing study with the full 125-patient cohort, being followed out to 2 years post-operation.

**Table 3:** Measurements of select lengths and areas on the rotator cuff tendon, and humeral orientation angles in a patient during 26 weeks following arthroscopic rotator cuff repair.

	Variable	0 week	3 week	12 week	26 week
**Tendon, mediolateral distances (mm)**	**V1T1**	11.0	16.3	23.3	29.4
	**V2T3**	17.5	20.9	32.7	40.8
	**V3T5**	23.9	26.7	36.3	41.5
	**V4T7**	21.5	23.0	33.6	38.5
**Tendon, antero-posterior lengths (mm)**	**T1T3**	7.5	9.5	12.2	14.1
	**T3T5**	12.4	13.2	12.7	11.4
	**T5T7**	9.0	11.1	14.6	12.9
**Tendon marker areas (mm**^**2**^**)**	**V1V2T3T1**	119.9	181.2	288.9	384.2
	**V2V3T5T3**	204.2	250.6	363.6	411.6
	**V3V4T7T5**	205.7	248.0	389.0	413.7
**Humeral orientation (degrees)**	**Extension**	2.3	4.6	2.0	4.0
	**External rotation**	28.4	56.4	59.1	56.9
	**Abduction**	17.2	9.0	5.2	0.3

## Discussion

The purpose of the current study was to describe a radiopaque tissue marker–its mechanical properties, biocompatibility and sub-chronic toxicity, proof-of-concept and finally clinical use for soft tissue localization and measurement in the context of rotator cuff repair. The results of the study demonstrate that the radiopaque marker possesses adequate handling characteristics and mechanical properties to readily allow fixation to tissues using standard open and arthroscopic knot-tying tools and techniques. Tensile properties of the radiopaque marker were comparable to 2–0 and 3–0 Prolene. The radiopaque marker also demonstrated biocompatibility and sub-chronic toxicity similar to polypropylene without barium sulfate; such minimal to mild inflammatory tissue reaction to implanted polypropylene has been reported in several studies, and suggests that barium sulfate in the radiopaque marker did not affect host response. No radiographic leaching, calcification or other significant local adverse events were observed following marker implantation.

Radiopaque markers implanted on the rotator cuff tendon and in the humerus (in the cadaver study) could be readily visualized under low-dose CT. Custom image analysis software was developed for registration of multiple CT image sets, definition of anatomic coordinate systems, placement of virtual markers, and computation of radiopaque marker locations and humeral rotation angles. In particular, software-generated “virtual” markers could be placed at anatomically relevant but otherwise technically challenging or even impossible physical locations. Virtual bone makers placed along the rotator cuff tendon footprint allow measurement of displacement of tendon-markers with respect to tendon footprint (e.g., V1T1), could provide information on tendon gapping or stretching at various locations along a repair/re-tear. Similarly, distance across the lateral row of tendon markers (T1T3 + T3T5 + T5T7) could provide information on the antero-posterior width of a repair/re-tear, and the area enclosed by the virtual and lateral tendon markers (V1V2T3T1 + V2V3T5T3 + V3V4T7T5) could provide information about the size of a repair/re-tear.

The cadaveric shoulder study showed that measurements using the radiopaque markers could be made with a precision of 0.66 mm for tendon lengths, 6.97 mm^2^ for areas, and 2.1° for humeral orientation angles. These values were relatively small compared to the respective absolute measures for this application (5–6% for lengths and areas, and 12% for humeral angles). In particular, the precision of length measurement was in the order of the marker size (~1mm knot diameter) and the resolution of the CT scanner (0.5 mm x 0.5 mm x 0.6 mm resolution), and would be considered acceptable for measuring rotator cuff tendon retraction after repair, which is on the order of centimeters^10^. Indeed, longitudinal data from the rotator cuff repair patient included for demonstrative purposes showed that medio-lateral lengths across the tendon repair (i.e., tendon retraction) increased 19 ±3 mm during 26 weeks post-operation. Concomitantly, areas across the rotator cuff tendon repair increased 227 ±3 mm^2^ during 26 weeks following repair.

Qualitative variability in arm position was shown to introduce ±3 mm of spurious variation in length measurements between rotator cuff tendon and bone markers [7]. Now to quantify variance in humeral orientation across multiple scans in a subject with the goal to possibly control for the associated measurement error, we designed our custom software to allow precise computation of humeral orientation with respect to the scapula. As expected, there was minimal variation in humeral orientation in the cadaver specimen that could be easily re-positioned between scans. In the clinical study, though attempts were made to consistently position the patient’s arm in a similar position during follow-up imaging, humeral orientation, particularly external rotation angle, was observed to vary as much as ~30° among scans in this patient. We will evaluate the influence of variation in arm position on error introduced in tendon length and area measurements as sufficient data becomes available in our ongoing patient cohort.[31]

The radiopaque tissue marker was developed to reduce technical challenges and risks associated with steel sutures [4–6] and tantalum beads [3, 8] that have previously been used for marking soft tissues. Affixing the steel suture/ tantalum bead markers to soft tissues is technically challenging and carries the risk of steel suture fragmentation [5], bead detachment and dislocation from the target site [3, 8]. While the current study demonstrated the utility of the radiopaque marker in rotator cuff repair, the marker could have several other applications and be placed on target tissues/organs through an open, percutaneous or endoscopic approach. For example, we have previously demonstrated that the radiopaque marker could be used to mark a ventral hernia defect as well as an implanted hernia mesh to monitor the change in defect size, mesh size, and hernia bulging using longitudinal CT scans in a porcine hernia repair model [23, 24]. We also envision the radiopaque markers could have application in other musculoskeletal surgical procedures like ACL or Achilles tendon repair or repair of the soft tissues following joint arthroplasty. Additionally we envision that the radiopaque marker could be used in marking a biopsy location or tumor resection bed, in tumor localization and monitoring tumor resection margins, in breast imaging, or vessel anastomosis. In addition to exploring the use of the radiopaque marker in a variety of applications, the potential utility of using the radiopaque tissue markers with dynamic imaging modalities such as biplanar radiography or RSA to assess joint kinematics [32, 33] or tissue strain [4, 5] should be assessed in future work.

The study’s objective was to demonstrate the proof-of-concept of using a radiopaque marker for visualization and measurement following surgical implantation. The marker is biocompatible and can be visualized under low-dose CT imaging (CTDIvol: 1.8 mGy/scan; effective dose: 1.0 mSv/scan). The health risks from radiation exposure of this magnitude are extremely small with no identifiable additional cancer risk [34, 35]. The patient example provided here demonstrates how software-based marker analysis of CT scans can allow for clinically relevant measurements to be made from the implanted markers, which in some applications may enhance the clinical utility of the markers over simple visual inspection. However, the marker is also readily visualized by standard planar X-ray and mammography as well as if radiographic visualization is all that is desired; the marker is visible on planar X-rays, even at viewing angles where it overlaps with bony structures, although it does have lower contrast compared to traditional tantalum markers.

## Conclusions

In summary, we report on a radiopaque tissue marker that could be readily affixed to tissues and implants and visualized under low-dose CT imaging. The marker is biocompatible and stable over time following implantation. Longitudinal CT image analysis of the radiopaque marker array implanted on the repaired rotator cuff could quantify variations in arm position and provide clinically-relevant length and area measurements with acceptable accuracy for measuring tendon retraction. We envision the radiopaque tissue markers to be useful for soft tissue localization and *in vivo* measurement of tissue and organ dimensions in potentially a broad range of surgical applications.

## Supporting information

S1 Checklist NC3Rs ARRIVE Guidelines checklist. (PDF)
